# Immunophenotype of myeloid granulocytes in Chinese patients with BCR::ABL1-negative myeloproliferative neoplasms

**DOI:** 10.1007/s10238-024-01363-7

**Published:** 2024-05-21

**Authors:** Fengting Liang, Xuelan Liang, Lingang Pan, Qianni Jin, Ju Deng, Minglin Hong, Wei Wei, Zhuanghui Hao, Huanying Ren, Hongwei Wang, Xiuhua Chen

**Affiliations:** 1 Key Laboratory of Molecular Diagnosis and Treatment of Hematologic Diseases of Shanxi Province, Taiyuan, China; 2 The Central Hospital of Wuhan, Wuhan, China

**Keywords:** Myeloproliferative neoplasm, Granulocyte, Immunophenotype

## Abstract

Typical BCR::ABL1-negative myeloproliferative neoplasms (MPN) are mainly referred to as polycythemia vera (PV), essential thrombocythemia (ET), and primary myelofbrosis (PMF). Granulocytes in MPN patients are involved in their inflammation and form an important part of the pathophysiology of MPN patients. It has been shown that the immunophenotype of granulocytes in MPN patients is altered. We used flow cytometry to explore the immunophenotype of MPN patients and correlate it with clinical parameters. The results showed that PMF patients and PV patients had higher CD15+CD11b+ granulocytes than ET patients and normal controls. When grouped by gene mutation, changes in the granulocyte immunophenotype of MPN patients were independent of the JAK2V617F and CALR mutations. There was no significant heterogeneity in immunophenotype between ET patients and Pre-PMF, and between Overt-PMF and Pre-PMF patients. Granulocytes from some MPN patients showed an abnormal CD13/CD16 phenotype with a significant increase in mature granulocytes on molecular and cytomorphological grounds, and this abnormal pattern occurred significantly more frequently in PMF patients than in ET patients. CD15–CD11b– was negatively correlated with WBC and Hb and positively correlated with DIPSS score, whereas high CD10+ granulocytes were significantly and negatively associated with prognostic system IPSS and DIPSS scores in PMF patients. In conclusion, this study demonstrates the landscape of bone marrow granulocyte immunophenotypes in MPN patients. MPN patients, especially those with PMF, have a significant granulocyte developmental overmaturation phenotype. CD10+ granulocytes may be involved in the prognosis of PMF patients.

## Introduction

BCR::ABL1-negative myeloproliferative neoplasms (MPNs) are clonal disorders originating from a single haematopoietic stem cell, mainly referred to as polycythaemia vera (PV), essential thrombocythaemia (ET) and primary myelofibrosis (PMF). PV is a monoclonal proliferative disorder of multipotent myeloid progenitor cells characterised by trilineage proliferation [1, 2]. It is characterised by elevated haematocrit and is associated with an increased risk of thrombotic events, leukaemic transformation and myelofibrosis (MF). ET manifests as megakaryocyte hyperproliferation leading to a markedly increased platelet count and is associated with thrombotic events, secondary myelofibrosis [3]. PMF, including pre-fibrotic/early PMF (pre-PMF) and overt fibrotic PMF (overt-PMF), is characterised by abnormal cytokine expression and myelofibrosis.The main clinical manifestations of PMF are severe anaemia, usually accompanied by hepatosplenomegaly, constitutional symptoms, thrombosis, haemorrhage, and transformation into acute myeloid leukaemia [4]. The common pathogenic mechanism of MPNs is through an acquired function mutation in one of the three disease driver genes (JAK2, MPL and CALR) without the need for other synergistic mutations to individually initiate and promote MPNs [5]. Approximately 10% of patients without JAK2V617F, CALR, or MPL mutations are known as “triple-negative” patients [6]. MPNs are uniformly referred to as myeloproliferative neoplasms because of their common clinical manifestations and pathogenic mechanisms and their ability to transform into each other.

Leucocytosis in MPN patients is mainly determined by the level of neutrophils, which are a risk factor for the development of thrombotic and haemorrhagic complications [7, 8]. It has been shown that angiogenic factors are generally enhanced in patients with MPN, but only in granulocytes with PMF, which exhibit JAK2V617F allele load dependence. Angiogenic factors of granulocytes were significantly reduced in MPN patients after hydroxyurea treatment [9]. Most of the altered markers in neutrophils were also found in CD34+ cells from patients with CALR and JAK2 mutations, but only a few genes showed similar expression patterns in both cell types [10]. Downstream of gene regulation, protein expression of integrin CD11b, tissue factor (TF) and leucocyte alkaline phosphatase were increased in JAK2V617F compared to CALR granulocytes [11–14]. In JAK2V617F MPN, neutrophil adhesion is increased by *β*1 integrin binding to VCAM1, and mouse models confirm these human observations that increased *β*1/*β*2 integrins are associated with venous thrombosis [15, 16]. These results suggest that granulocytes from MPN patients are involved in the pathophysiology of MPN and play an important role in MPN disease progression.

Flow cytometry is widely used in the diagnosis and determination of prognosis of haematological diseases due to its ability to analyse tumour phenotypes and highlight specific tumour-associated antigen profiles [17]. For example, the assessment of minimal residual disease (MRD) has increasingly become a cornerstone of the clinical management of haematological malignancies. Myeloid cell immunophenotyping is an important tool for the diagnosis of myeloid dysplasia in MDS [18], which can be accompanied by a variety of myeloid cell immunophenotypic abnormalities characterised by abnormal granulocyte CD13/CD16 phenotypes, abnormal expression of CD56 by monocytes and decreased expression of CD71 by erythrocytes [19, 20]. It has been shown that monocytes in patients with MPN abnormally express CD56 and have an increase in intermediate and non-classical monocytes [21], furthermore, granulocytes in patients with PMF also show abnormal features such as an abnormal CD13/CD16 phenotype and expression of CD56 [22]. Meanwhile, the use of multiparametric flow cytometry has helped to refine the current prognostic stratification model for myelofibrosis [23]. In view of these premises, this research applied flow cytometry to detect the immunophenotypic characteristics of granulocytes and their changes in patients with MPN, as well as to investigate the clinical significance of these changes.

## Methods and materials

### Subject

Patients with a confirmed diagnosis of MPN from January 2021 to December 2023 in our center were collected. Patients are further enrolled in this study if patients with MPN were not treated with ruxolitinib or other JAK2 inhibitors at the time of initial diagnosis or at the time of diagnosis, or patients had been treated with ruxolitinib but had not been treated with ruxolitinib in the last six months or more (with concomitant JAK2V617F positivity). Clinical information of patients were collected by medical records, including age, sex, blood count, bone marrow pathology and morphology, genetic testing information, biochemical tests and medical history. A total of 22 healthy controls without haematological disease were also included. Informed consent was obtained from all participants. Baseline characteristics of MPN patients and healthy controls are shown in Table 1.

**Table 1:** Baseline characteristics of the study population

	PV	ET	PMF	Control
N	36	41	51	22
Age (median, range)	61 (34, 93)	59 (13, 84)	67 (45, 84)	47 (22, 83)
Sex				
Female	16	23	27	10
Male	20	19	14	12
Mutation types				
JAK2V617F	30	29	33	–
CALR	0	10	7	–
MPL	0	0	5	–
JAK2 exon12	0	0	0	–
Triple-negative	3	2	5	
Undetermined	3	0	1	–
Newly diagnosed cases	29	39	40	–
Treatments				
Hydroxyurea	6	1	2	–
Ruxolitinib	–	–	5	–
Interferon	–	1	–	–

### Flow cytometry

Take 8 flow-through tubes labelled 1–8 and add the following 8 antibody combinations to each tube: CD7-FITC, CD117-PE, CD19-PC5, CD45-PC7; CD38-FITC, CD34-PE, CD123-PC5, CD45-PC7; CD16-FITC, CD13-PE, CD33-PC5, CD45-PC7; CD15-FITC, CD11b-PE, CD34-PC5, CD45-PC7; CD64-FITC, CD14-PE, HLA-DR-PC5, CD45-PC7; CD36-FITC, CD56-PE, CD38-PC5, CD45-PC7; CD71-FITC, CD235a-PE, CD34-PC5, CD45-PC7; CD10-FITC, CD19-PE, CD34-PC5, CD45-PC7, and the dosage of each antibody was 5 µl. For each tube, 50 µl of fresh bone marrow was added and incubated at room temperature for 30 min before the red colour was broken. For the cytoplasmic antigen tube, CD45-PC7 and bone marrow were added and incubated for 30 min, and then Buffer 1 (50 µl) was added and fixed for 15 min, followed by the addition of Buffer 2 and cytoplasmic antibodies, such as cMPO and cCD79a, and incubation for 20 min. All antibodies and reagents were purchased from BD Company (USA).

SSC/CD45 was applied to classify bone marrow cells into lymphocytes (L), monocytes (M), granulocytes (G), primitive cells (B) and nucleated cells (E). When labelling individual antigens, the rate of monocyte or granulocyte individual antigen positivity (%) was counted using CD45 as the y-axis, individual antigens as the x-axis and based on the G-gate. Because MFI is susceptible to instrumental instability and because we collected cases over a large time span, we recorded only the percentage (%) of positivity for a single antigen or for both antigens. The detailed gating strategy is shown below.

### Gene mutation detection

Gene mutations were sequenced using one generation sequencing. After DNA samples were extracted from the patient’s bone marrow, DNA libraries were constructed according to the instructions. Genome sequencing was done on the Illumina Miseq platform (San Diego, CA, USA), and the gene sequencing panel contained currently known MPN driver genes, such as JAK2V617F, CALR 1, CALR 2, and MPL W515.

### Bone marrow pathology

Biopsies were at least 1.5 cm long and paraffin-embedded, and sections were 3–4 μm thick. Bone marrow biopsies were stained with cytochemical stains including routine HE and/or Giemsa, reticulofibrillar (silversmith) stain, glycogen (PAS) stain, chloroacetic acid AS-D naphthol esterase stain (CE), and Prussian blue stain (iron-stain) and analysed under light microscope.

### Data analysis

GraphPad Prism software (version 8.3.0, GraphPad Software, La Jolla CA) was used for statistical analyses and graphing, while IBM SPSS Statistics 25 was used for proofreading. Dunn’s multiple comparison test was used for non-normally distributed data in three or more groups, and the Mann–Whitney test was used to compare data between two groups. When correlation analyses were required, all were analysed uniformly for significance using non-parametric Spearman’s correlation. *P* < 0.05 was considered statistically significant. *P < 0.05; **P < 0.01; ***P < 0.001; ****P < 0.0001.

## Results

### Comparison of single and dual antigen combinations in different MPN patients and HD granulocytes

**Fig. 1:**
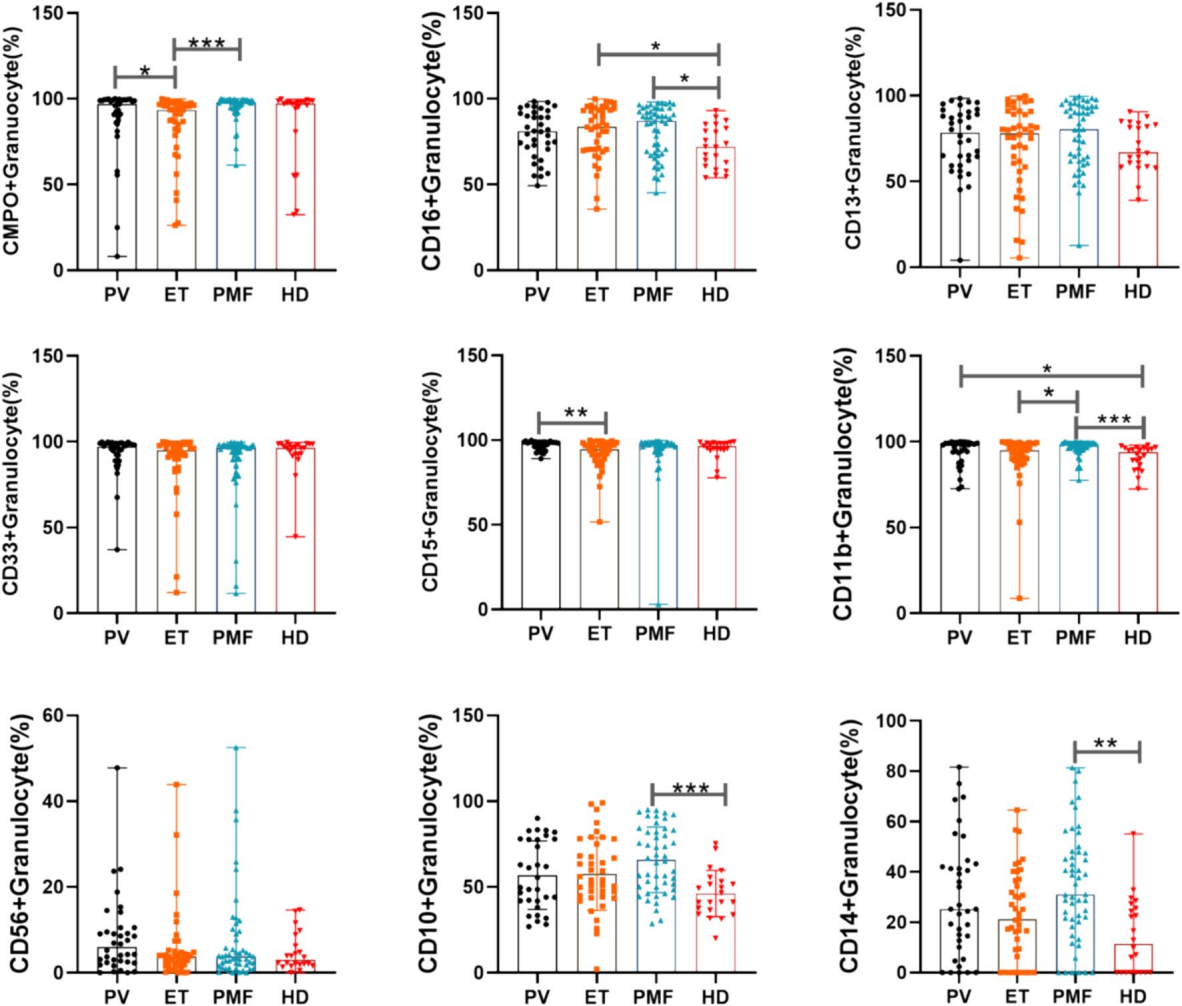
Comparison of individual antigens in MPN patients and healthy controls. Individual patients may lack data for a particular antigen

Comparison of individual antigens allows initial judgement of the individual molecular markers that help to differentiate MPN. As shown in Fig. 1, compared to normal controls, PMF patients and PV patients highly expressed mature granulocyte-associated antigens such as CD16 and CD11b, but PMF patients also highly expressed CD10 (which is expressed at the segmented granulocyte stage). In contrast, ET patients did not show these changes and even had lower CD11b expression than PMF patients. These results suggest that granulocyte maturation is higher in patients with PMF. In addition, compared to controls, granulocytes from PMF patients also highly expressed CD14. cMPO expression was lower in ET patients than in PV and PMF patients, while CD15 expression was lower in ET patients than in PV patients. Other surface antigens were not statistically different between MPN patients and normal controls.

**Fig. 2:**
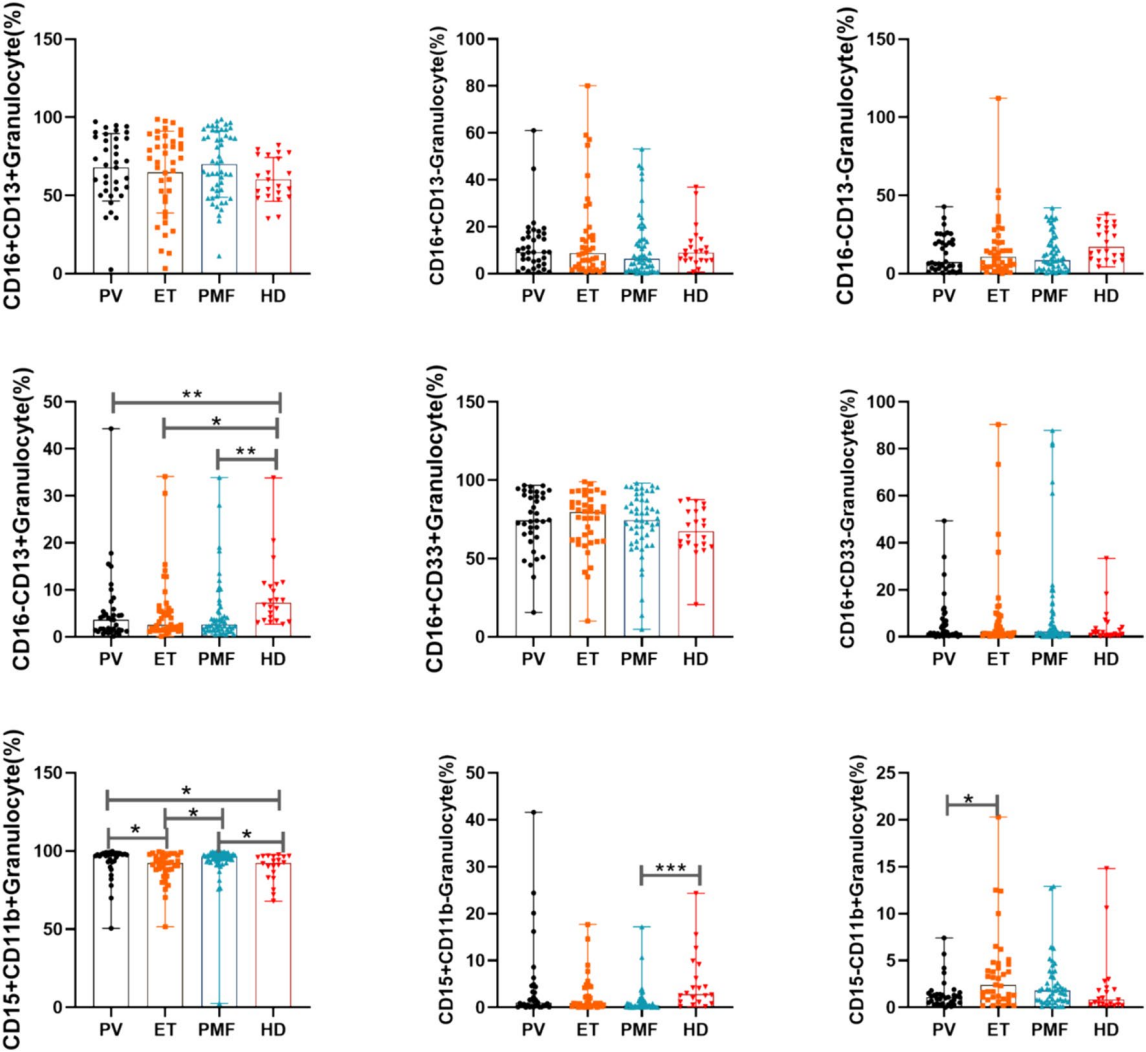
Dual antigen combinations of granulocytes from MPN patients and controls

Combinations of two or more antigens can initially identify subpopulations of cells with different functions or determine cell development. As shown in Fig. 2, CD16–CD13+ naïve granulocytes were generally lower in MPN patients than in normal controls. On the other hand, PMF patients and PV patients had higher CD15+CD11b+ granulocytes than ET patients and normal controls. The results of combining single and double antigens illustrated some heterogeneity in the immunophenotype of bone marrow granulocytes from MPN patients with different disease entities. Bone marrow granulocytes from PMF patients highly expressed antigenic molecules such as CD10, CD16 and CD14, and their granulocyte maturation was increased.

### Immunophenotyping of bone marrow granulocytes in patients with ET, Pre-PMF, and PMF

**Fig. 3:**
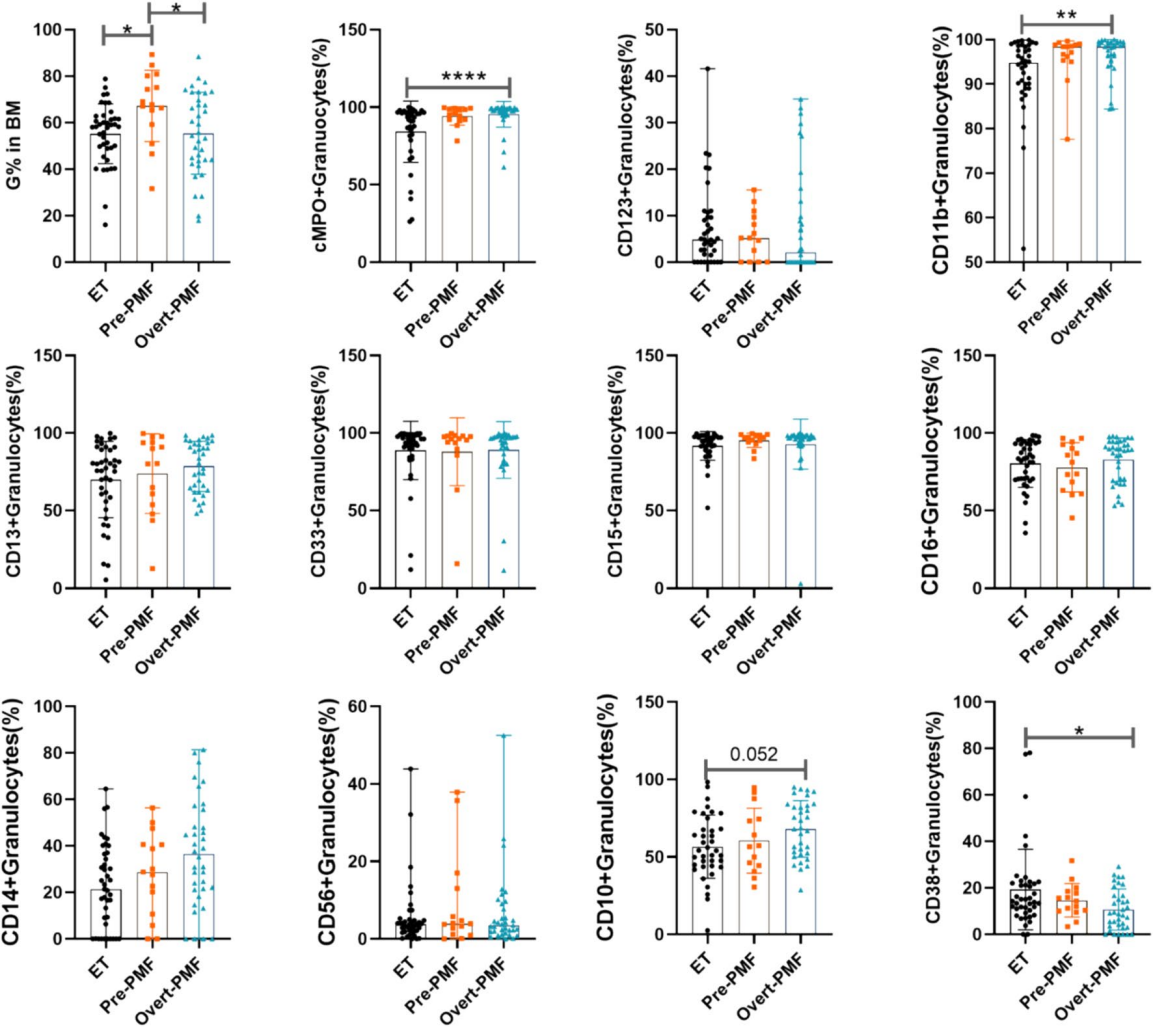
Comparison of granulocyte immuneophenotypes in patients with ET and Pre-PMF and Overt-PMF. 15 patients with Pre-PMF and 36 patients with Overt-PMF

Further comparisons of granulocyte immunophenotypes in ET, Pre-PMF, and Overt-PMF patients were made to explore molecular markers that would be useful in distinguishing between ET and Pre-PMF patients or the immunophenotypes of granulocytes in patients with different stages of PMF. As shown in Fig. 3, the percentage of bone marrow granulocytes was significantly higher in Pre-PMF patients than in ET and Overt-PMF patients. Although other antigens were not significantly different between ET and Pre-PMF patients and between Pre-PMF and Overt-PMF patients, bone marrow granulocyte expression of CD11b, cMPO remained significantly higher in Overt-PMF patients than in ET patients. These results suggest that there is no significant heterogeneity in granulocyte immunophenotypes between Pre-PMF patients and Overt-PMF patients, but the immunophenotypes of ET patients and Overt-PMF patients remain heterogeneous.

### Granulocyte abnormal phenotype and aberrant expression of CD56 in some MPN patients

**Fig. 4:**
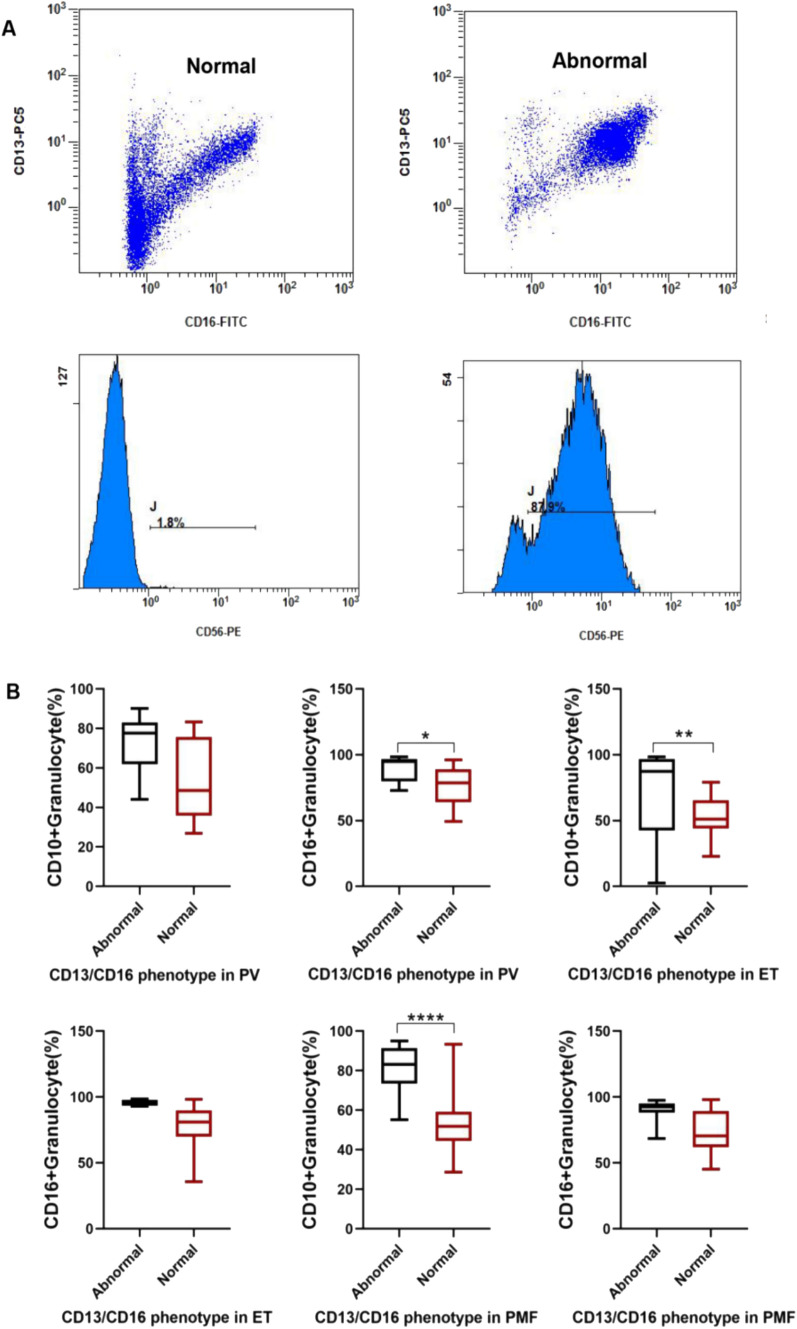
**A** Graphical representation of the CD13/CD16 phenotype in a case of HD and a case of MPN and a histogram of CD56. **B** Statistical differences comparing CD10 or CD16 in MPN patients with normal CD13/CD16 phenotype and abnormal CD13/CD16 phenotype, respectively

Granulocyte development in patients with MPN can be initially determined using the abnormal CD16/CD13 phenotype. Among the 128 MPNs, approximately 43% (22/51) of PMF patients, 16.7% (7/41) of ET patients, and 19.4% (7/36) of PV patients developed CD13/CD16 phenotypic abnormalities (Fig. 4A). In contrast, the frequency of CD13/CD16 phenotypic abnormalities was significantly higher in PMF patients than in ET patients (*P* = 0.016). Analysis of the immunophenotype of granulocytes from patients with an abnormal CD13/CD16 phenotype and patients with a normal CD13/CD16 phenotype revealed that granulocytes from patients with an abnormal CD13/CD16 phenotype expressed high levels of mature granulocyte-associated antigens such as CD16, CD10 and other antigens (Fig. 4B). These results suggest the presence of in the granulocytes of MPN patients with a predominantly excessive increase in mature granulocytes. In addition to the abnormal CD13/CD16 phenotype, granulocytes abnormally expressed CD56 in approximately less than 8% of MPN patients (Fig. 4).

### Correlation analysis of granulocyte immunophenotype and bone marrow cell morphology in MPN patients with granulocytes at different periods of time

**Fig. 5:**
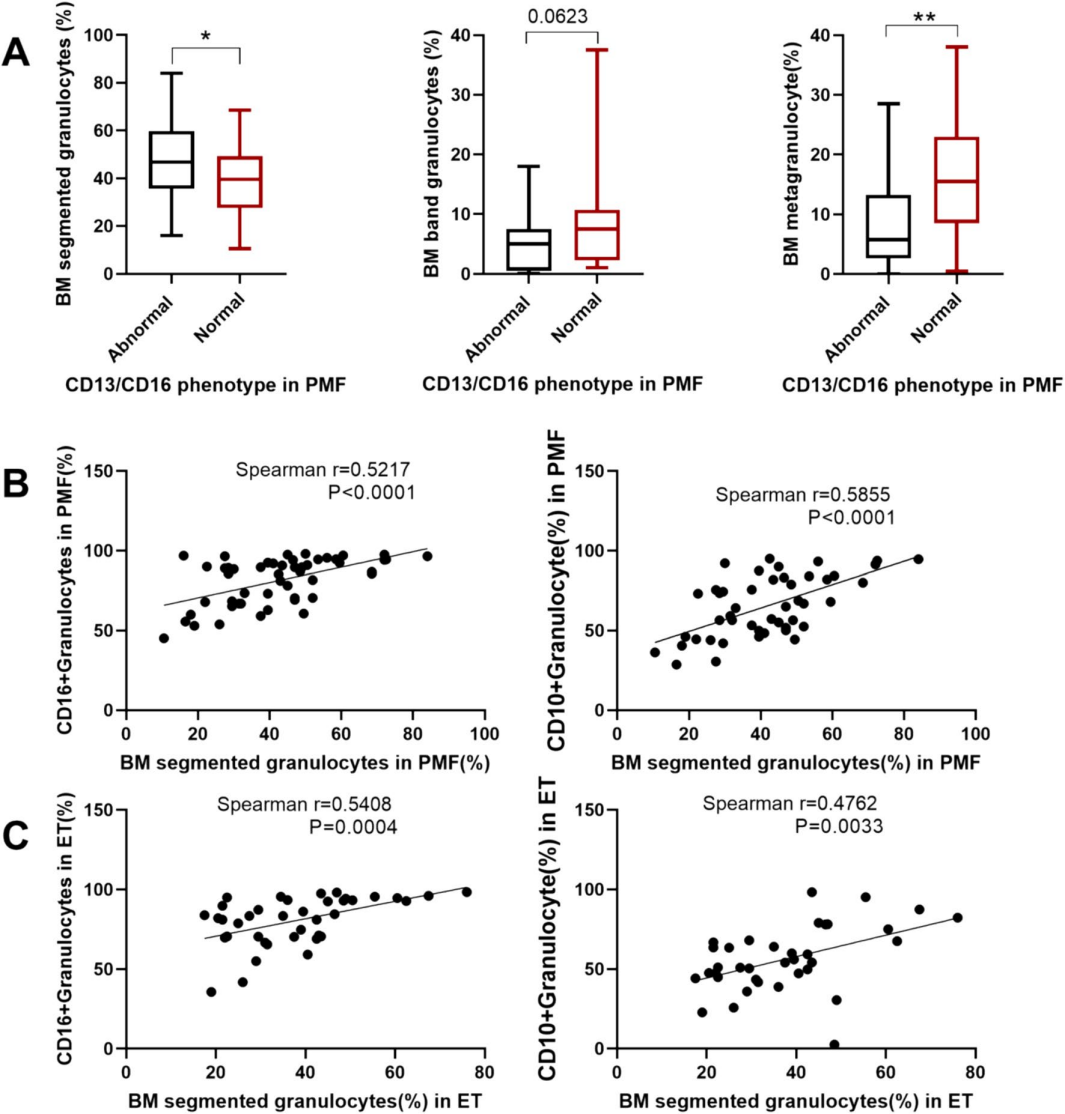
**A** Comparison of the proportions of cytomorphologically segmented granulocytes, band granulocytes and metagranulocytes to the whole bone marrow in patients with PMF with abnormal CD13/CD16 phenotype and patients with PMF with normal CD13/CD16 phenotype, respectively. **B** Analysis of the correlation between the proportion of segmented granulocytes to whole bone marrow and CD16 or CD10 on bone marrow cell morphology in patients with PMF. **C** Analysis of the correlation between the proportion of segmented granulocytes to whole bone marrow and CD16 or CD10 on bone marrow cell morphology in patients with ET

To further validate the reliability of the results, bone marrow cytomorphology was analysed for the relationship between the proportion of granulocytes, band granulocytes and metagranulocytes to the whole bone marrow and the bone marrow granulocyte immunophenotype in MPN patients. As shown in Fig. 5A, PMF patients with abnormal CD13/CD16 phenotype had significantly higher cytomorphological segmented granulocytes than PMF patients with normal CD13/CD16 phenotype. On the other hand, PMF patients with abnormal CD13/CD16 phenotype had significantly lower cytomorphology of metagranulocytes than PMF patients with normal CD13/CD16 phenotype. In addition, as shown in Fig. 5B and C, CD10 and CD16 were significantly and positively correlated with the percentage of cytomorphologically lobulated nucleated granulocytes in MPN patients. These results further suggest that, both molecularly phenotypically and cytomorphologically, the granulocyte abnormal phenotype present in some MPN patients is characterised by an excessive increase in mature granulocytes.

### Changes in bone marrow granulocyte immunophenotype in MPN patients with different mutational backgrounds

**Fig. 6:**
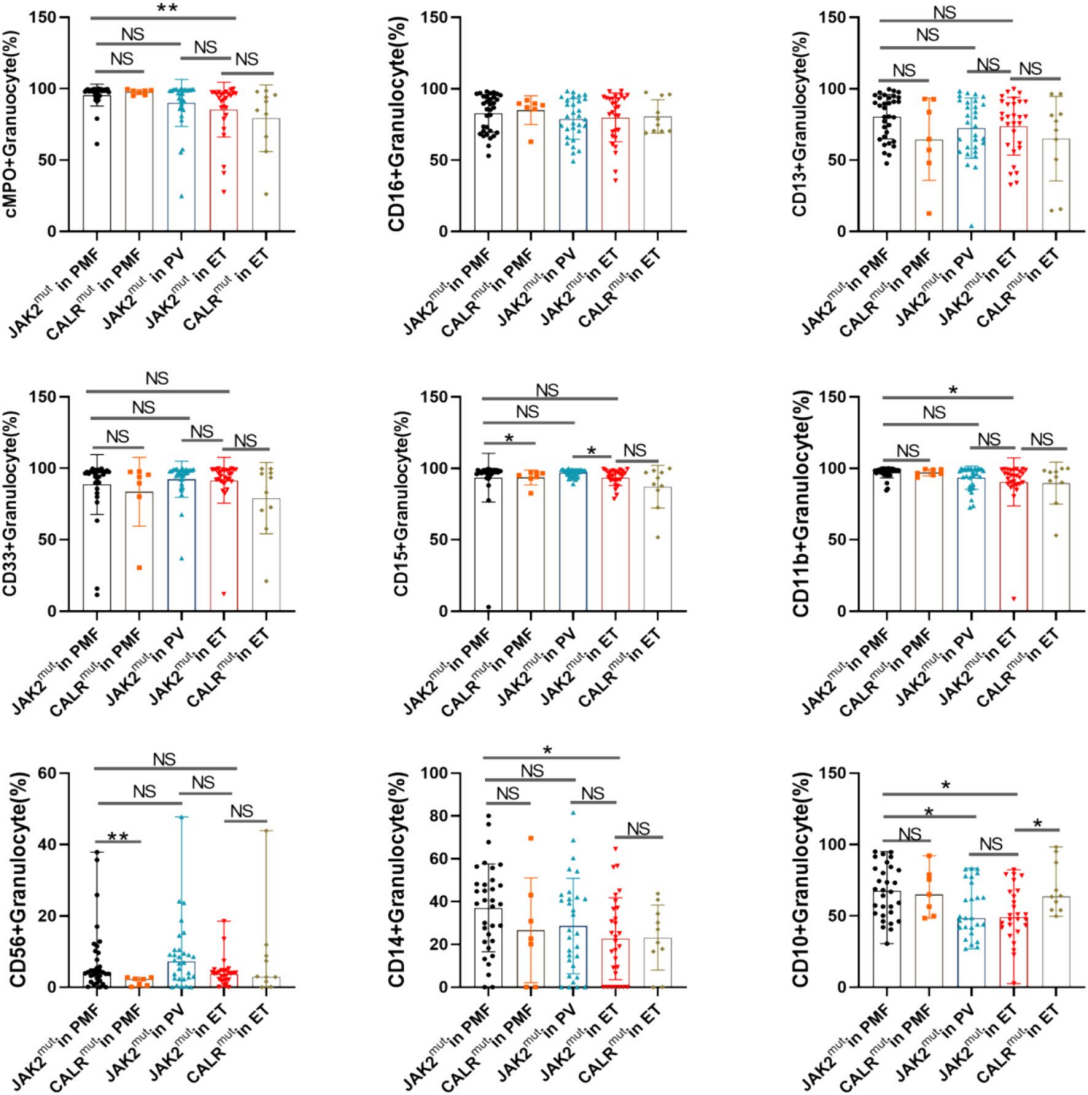
Comparisons were made only between patients with concomitant JAK2 mutations or between different mutations in the same disease. JAK2^mut^ refers to the JAK2V617F mutation. JAK2^mut^ in PMF, JAK2^mut^ in PV and JAK2^mut^ in ET were compared using Dunn’s multiple comparison method. The Mann Whitney test was used to compare the two groups. *P* < 0.05 was considered statistically significant. NS indicates no statistical significance. CD16+ granulocytes were not statistically significant between groups

To investigate whether mutations are important factors in the altered immunophenotype of granulocytes in patients with MPN, we compared the immunophenotypes of granulocytes from patients with different diseases for the same mutated gene or from patients with different mutational backgrounds for the same disease. As shown in Fig. 6, when the immunophenotypes of granulocytes from MPN patients accompanied by the JAK2V617F mutation were compared, the expression of cMPO in ET patients remained lower than that of PMF patients, while the expression of CD15 in granulocytes from ET patients remained lower than that of PV patients. In addition, granulocyte expression of CD10 was significantly higher in PMF patients with JAK2V617F mutation than in ET and PV patients with concomitant JAK2V617F mutation. When comparing the immunophenotypes of granulocytes from patients with different mutant genes in patients with ET or PMF, respectively, an immune antigen that was statistically different between patients with JAK2V617F-mutated PMF and patients with CALR-mutated PMF was not detected as well as a statistically different immunoantigen between patients with JAK2V617F-mutated ET and patients with CALR-mutated ET. These results suggest that JAK2 and CALR mutations are not important causes of altered granulocyte immunophenotypes in MPN patients. On the other hand, when stratifying MPN patients by mutations, the heterogeneity of granulocyte immunophenotypes in MPN patients with different disease entities remains. PMF patients with concomitant JAK2V617F mutation had significantly higher granulocyte maturation than ET or PV patients.

### Analysis of the correlation between granulocyte immunophenotype and clinical parameters in MPN patients

The relationship between clinical parameters of PMF patients and their granulocyte immunophenotype was further investigated. The myelofibrosis grades in PMF patients was classified as MF-0, MF-1, MF-2, and MF-3 according to WHO (2016) criteria and further evaluated. As shown in Table 2, the bone marrow granulocyte ratio was positively correlated with WBC, Hb, PLT, and JAK2V617F mutations, whereas it was negatively correlated with myelofibrosis grade. CD11b+ granulocytes were negatively correlated with circulating blasts > 1% in PMF patients and with the PMF International Prognostic System DIPSS score. CD15–CD11b– was negatively correlated with WBC and Hb and positively correlated with DIPSS score. CD10+ was positively correlated with PLT count and negatively correlated with IPSS score and DIPSS score. These results suggest that the increase in bone marrow naïve granulocytes and the decrease in mature granulocytes in PMF patients are associated with a variety of poor prognostic factors, such as circulating blasts > 1%, IPSS, and DIPSS scores in PMF patients.

**Table 2:** *P*-values for comparison of the correlation between the proportion of different granulocyte subpopulations in the bone marrow and clinical characteristics of PMF patients

	G%	CD11b+	CD15+CD11b−	CD15−CD11b−	CD10+	CD56+	CD16+
Sex; man	0.970	0.275	0.855	0.115	0.815	0.0005 *r* = 0.476	0.635
Age	0.163	0.051 *r* = 0.277	0.093	0.078	0.809	0.705	0.142
Circulating blasts > 1%	0.553	0.038 *r* = − 0.293	0.006 *r* = 0.378	0.083	0.148	0.472	0.258
Constitutional symptoms	0.620	0.328	0.189	0.679	0.293	0.149	0.497
Splenomegaly	0.232	0.872	0.914	0.642	0.532	0.858	0.958
WBC count (× 109/L)	< 0.0001 *r* = 0.679	0.258	0.554	0.029 *r* = − 0.308	0.304	0.619	0.781
Hb (g/L)	< 0.0001 *r* = 0.527	0.078	0.135	0.0045 *r* = − 0.395	0.182	0.962	0.357
PLT (× 1012/L)	0.0084 *r* = 0.365	0.097	0.011 *r* = − 0.355	0.102	0.018 *r* = 0.339	0.111	0.099
LDH(U/L)	0.875	0.864	0.350	0.970	0.196	0.920	0.464
Bone marrow fibrosis grade	0.048 *r* = − 0.278	0.713	0.757	0.777	0.736	0.899	0.918
JAK2V617F mutation	0.009 *r* = 0.365	0.014 *r* = 0.348	0.160	0.0003 *r* = − 0.491	0.174	0.213	0.273
CALR mutation	0.290	0.815	0.825	0.168	0.908	0.009 *r* = − 0.365	0.989
MPL mutation	0.337	0.183	0.719	0.183	0.433	0.352	0.242
IPSS Score	0.477	0.184	0.073	0.520	0.012 *r* = − 0.410	0.145	0.377
DIPSS score	0.155	0.042 *r* = − 0.292	0.028 *r* = 0.314	0.036 *r* = 0.301	0.012 *r* = − 0.362	0.185	0.327

Statistical analyses were performed using Spearman’s correlation analysis, and “*r*” refers to “Spearman *r*”

The correlation between clinical parameters of patients with PV or ET and their granulocyte immunophenotype was also investigated. As shown in Table 3, the proportion of bone marrow granulocytes in PV patients was significantly and positively correlated with their WBC counts, whereas other granulocyte subpopulations had no significant correlation with clinical parameters such as age, splenomegaly, WBC, PLT, and Hb in ET patients. As shown in Table 4, the proportion of bone marrow granulocytes in ET patients showed a significant positive correlation with their WBC counts and Hb. Bone marrow CD15-CD11b-granulocyte subpopulations in ET patients showed a positive correlation with Hb.

**Table 3:** *P*-values for comparison of the correlation between the proportion of different granulocyte subpopulations in the bone marrow and clinical characteristics of PV patients

	G%	CD11b+	CD15+CD11b−	CD15−CD11b−	CD10+	CD56+	CD16+
Age	0.311	0.979	0.933	0.395	0.903	0.128	0.624
Splenomegaly	0.535	0.812	0.965	0.337	0.737	0.329	0.679
WBC count (× 10^9^/L)	0.0002 *r* = 0.580	0.428	0.531	0.702	0.355	0.689	0.978
Hb (g/L)	0.483	0.567	0.977	0.899	0.361	0.174	0.244
PLT (× 10^12^/L)	0.562	0.270	0.417	0.184	0.847	0.08	0.251

Statistical analyses were performed using Spearman’s correlation analysis.* P* < 0.05 was significant

**Table 4:** *P*-values for comparison of the correlation between the proportion of different granulocyte subpopulations in the bone marrow and clinical characteristics of ET patients

	G%	CD11b+	CD15+CD11b−	CD15−CD11b−	CD10+	CD56+	CD16+
Age	0.656	0.734	0.818	0.575	0.137	0.134	0.785
Splenomegaly	0.775	0.985	0.577	0.716	0.608	0.435	0.831
WBC count (× 109/L)	0.0022 *r* = 0.476	0.205	0.361	0.409	0.428	0.212	0.725
Hb (g/L)	0.048 *r* = 0.318	0.089	0.497	0.042 *r* = 0.349	0.156	0.255	0.500
PLT (× 1012/L)	0.994	0.987	0.664	0.085	0.243	0.931	0.983

Statistical analyses were performed using Spearman’s correlation analysis. *P* < 0.05 was statistically significant

## Discussion

In normal biological processes, some cell surface antigens appear or disappear in a certain order as primitive, naïve, and mature cells gradually differentiate. CD33 begins to be stably expressed in naïve granulocytes. CD16 and CD11b cells are stably expressed by cells at late juvenile and following stages. CD10 is stably expressed in segmented granulocytes. However, with physiopathological changes, the patient’s granulocytes may show a lack of original antigenic expression or abnormal antigenic expression, which may also be accompanied by an abnormal antigenic expression pattern. Abnormal expression of CD56 by monocytes and granulocytes has been reported in MPN [21, 22], and granulocytes in PMF patients also develop an abnormal CD13/CD16 phenotype [22]. Our results suggest that some PV and ET patients also develop CD13/CD16 phenotypic abnormalities, and that the frequency of CD13/CD16 phenotypic abnormalities is significantly higher in PMF patients than in ET patients. This CD13/CD16 phenotypic abnormality is a manifestation of an over-abundance of mature granulocytes predominantly expressing mature granulocyte-associated antigens such as CD16 and CD10. MDS patients are also associated with abnormal granulocyte CD13/CD16 phenotype, but this abnormality is a predominantly reduced CD16 expression phenotype. Therefore, the abnormal granulocyte CD13/CD16 phenotype in MPN patients is significantly different from that of MDS. In addition, granulocytes in MPN patients also highly express CD56, but in less than 10% of cases.

CD56, also known as neural cell adhesion molecule 1 (NCAM1), is usually highly expressed in natural killer (NK) cells. In addition, NCAM1 expression has been found in rare subpopulations of T and B lymphocytes, dendritic cells and neural or mesenchymal stem cells [24, 25]. Aberrant expression of CD56 is also present in AML patients and is associated with reduced complete remission rates, increased relapse rates and reduced overall survival [26]. Recent studies have shown that aberrant expression of CD56 in AML patients may be involved in the maintenance of leukaemia stem cells and confer resistance through activation of the MAPK pathway [27]. CD56+ granulocytes are significantly increased not only in haematological disorders, such as MDS and CML [28], but have also been reported in PMF patients. However, its clinical significance in PMF patients is not clear. Our study showed that abnormal expression of CD56 by myeloid cells in MPN patients was uncommon, and about less than 10% of MPN patients had abnormally high granulocyte expression of CD56. High granulocyte expression of CD56 in PMF patients was associated with CALR mutations. In contrast, monocyte expression of CD56 in PMF patients did not show a significant correlation with clinical parameters and is therefore not shown.

As mentioned previously, MPN is thought to result from hyperactivation of the JAK/STAT signalling pathway through mutations in driver genes such as JAK2V617F, CALR, and MPL [29]. The JAK/STAT signalling pathway widely mediates a variety of biological processes such as cell proliferation, differentiation, migration, apoptosis, and immune regulation [30]. Therefore, we hypothesised that the altered immunophenotype of myeloid cells in MPN patients may be due to overactivation of the JAK/STAT pathway as a result of the JAK2V617F mutation. However, our findings suggest that the granulocyte immunophenotypes of MPN patients are not associated with the JAK2V617F mutation and the CALR mutation. Moreover, MPN patients with JAK2V617F mutation still showed heterogeneous expression of the granulocyte immunophenotype.

To further explore the clinical significance of the immune phenotype of MPN patients, we combined MPN patients with a variety of clinical data. Since PMF patients are a poor prognostic type of MPN patients, our study focused on the correlation analysis of the clinical parameters of PMF patients with their autoimmune phenotype. From 2010 to date, several PMF prognostic systems have been widely recognised internationally. The 2010 IPSS and DIPSS prognostic systems used clinical indicators such as patients with circulating mother cells > 1%, WBC > 25 × 10^9^/L, Hb < 100 g/L, and age > 65 years as risk factors for prognostic modelling [31, 32]. With the development of molecular testing, MPN driver genes have been shown to correlate with prognosis. Studies have shown that CALR mutations favour overall survival in PMF patients, while MPL mutations are not detrimental to overall survival [33, 34]. In addition to driver mutations, undetected MPN driver mutations and myelofibrosis are independent risk factors for survival in PMF patients [35, 36]. On the other hand, with the update of the PMF prognostic system, non-driver mutations such as ASXL1, EZH2, SRSF2, IDH1/2 and other genetic variants in PMF patients are also high-risk factors affecting PMF patients [37]. Since most of the patients in our clinical data were not tested for MPN non-driver genes, only the IPSS score and DIPSS score were used as prognostic risk factors for PMF patients.

Our results showed that high expression of CD11b and CD10 in granulocytes from PMF patients was negatively correlated with the IPSS and DIPSS prognostic system scores, whereas high expression of CD15+CD11b− and CD15–CD11b– naïve granulocyte subpopulations was positively correlated with the DIPSS score. Recent studies have shown that CD10 distinguishes mature granulocytes from immature granulocytes better than CD16 and CD11b [38]. These results imply that the increase in bone marrow naïve granulocytes and the decrease in mature granulocytes in PMF patients may be associated with a poor prognosis.

In conclusion, our data show that there is heterogeneous expression of the granulocyte immunophenotype in MPN patients. Some patients with MPN have dysplastic or abnormal granulocyte development. This study demonstrates the landscape of bone marrow granulocyte immunophenotypes in MPN patients and explores their potential clinical significance, as well as providing further insights into the immune microenvironment based on granulocyte immune antigens in the pathophysiology of MPN.

## Data Availability

The authors confirm that the data supporting the findings of this study are available within the article.
